# Self‐Cleaning Integrative Aerogel for Stable Solar‐Assisted Desalination

**DOI:** 10.1002/gch2.202000063

**Published:** 2020-12-16

**Authors:** Yufei Gu, Xiaojiang Mu, Pengfei Wang, Xiaoyang Wang, Yongzhi Tian, Anyun Wei, Jiahong Zhang, Yulian Chen, Zhiqiang Sun, Jianhua Zhou, Lei Miao

**Affiliations:** ^1^ Guangxi Key Laboratory of Information Material Guangxi Collaborative Innovation Center of Structure and Property for New Energy and Materials School of Material Science and Engineering Guilin University of Electronic Technology Guilin 541004 China; ^2^ Department of Materials Science and Engineering SIT Research Laboratories Innovative Global Program Faculty of Engineering Shibaura Institute of Technology Tokyo 135‐8548 Japan

**Keywords:** integrative evaporators, salt rejection, solar‐assisted desalination generators, solar steam generation

## Abstract

The solar‐assisted desalination generator (SADG) shows great potential for solving water scarcity problems. However, salt precipitation and accumulation is still a challenge for SADG, which slows down solar steam generation performance of evaporator during operation. Here, a facile integrative evaporator featuring stable and high evaporation performance breaks this bottleneck. By using a rational design in which amorphous carbon particles are evenly composited within the porous chitosan aerogel, the evaporator not only integrates excellent light absorption, heat management, and water transportation abilities but also endows a large vapor escape space. Upon desalination, salt concentration ingredients between carbon particles and chitosan membranes can be quickly balanced by water transport in interconnected chitosan chains, and thus salt precipiation on the evaporator surface would be avoided. Compared to other salt‐rejection evaporators, the integrative evaporator can operate in 3.5 wt% brine for 60 days without salt precipiation and exhibits a stable evaporation rate (1.70 kg m^−2^ h^−1^), indicating its potential for practical applications in seawater desalination and the harvest of clean drinking water.

## Graphical abstract

An integrative aerogel is fabricated by a facile freeze‐drying method. During solar‐assisted desalination, the interconnected chitosan networks of the aerogel balances the salt concentration gradient between particles and chitosan skeleton depending on the effect of solar‐driven water transportation. The generator has stable solar‐assisted desalination performance with an evaporation rate of 1.70 kg m^−2^ h^−1^ under one‐sun illumination for 60 days in 3.5 wt% brine.

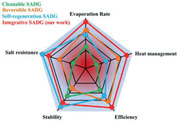

## 1 Introduction

Water scarcity has been cited as one of the most rigorous global problems for human. To solve the issue, man demands fresh water from the sea. Reverse osmosis has been industrialized as a recognized desalination technology, however, the hugeconstruction and maintenance costs make the technology difficult to popularize. In recent years, solar steam generation as a new emerging solar‐assisted desalination technology, it has a great potential to solve water and energy shortages due to high photothermal conversion efficiency, low‐cost, and sustainability.^[^ ^1^, ^2^ ^]^ Typical configuration for a solar steam generator (SSG) is efficient light absorber which converts incident sunlight to heat energy and heats water to generate vapor, while bulk water is pumped continuously by a transport layer. However, in a long‐term solar‐assisted desalination process, the fast gas‐liquid exchange at the air‐liquid interface of the absorber layer leads to a significant increase in the local saline concentration.^[^ ^3^ ^]^ When the salt concentration at the surface of SSG is up to saturate, the salts will preferential precipitate and accumulate at the interface, resulting in dramatical desalination performance degradation. Moreover, for a long‐term desalination, the stability of materials needs to be rigorously tested, which involves not only the stable of SSG itself, but also the chemical stability of photothermal materials. Because desalination is operated under a high humidity and high salinity condition, wet atmosphere corrosion is inevitable when SSG consists of metal matrix, and if possible, materials harmful to the environment should be avoided.^[^ ^4^ ^]^ Thus SSG with high regeneration ability, stability, and environmental‐friendly in desalination is a new tendency.^[^ ^5^ ^]^

Currently, the solar‐assisted desalination generator (SADG) reported can be divided into three types. The cleanable SADG consists of photothermal materials and polymer film, which exhibits high reusable ability due to its flexible and stable structure.^[^ ^6^, ^7^, ^8^, ^9^, ^10^, ^11^ ^]^ For this SADG, the addition of salt removal process would decrease the efficiency of solar‐assisted desalination and increase the operating costs. The reversible SADG consists of solar absorber and functional layer, in which the functional layer is hydrophilic material (such as cotton, melamine sponge, and polyvinyl alcohol gel).^[^ ^12^, ^13^, ^14^, ^15^, ^16^, ^17^, ^18^, ^19^, ^20^, ^21^, ^22^, ^23^, ^24^, ^25^, ^26^ ^]^ During long‐term solar desalination, the functional layer can transport water to the absorber for vaporing, and reduce the heat conduction from the absorber to bulk water, as well as maintain the dynamic salt concentration level below the salt saturation in the system. However, the salt adjustment capacity of functional layer is weak. The evaporation rate still decreases in long‐term solar desalination because the fast gas‐liquid exchange at the surface of absorber layer makes it achieve salt saturation easily. To be sure, salt at the surface of the SADG will disssolve spontaneously in hours without solar illumination, which partially meet the practical sustainable demand of solar desalination.

The self‐regeneration SADG can desalinate seawater without salt precipitation for a long time and keep a steady evaporation rate, which is based on the optimization and improvement of the internal structure of the reversible SADG. Hu and co‐workers reported a salt‐rejecting SADG that demonstrated the immobilization of graphene‐based material on hydrophobic polytetrafluoroethylene (PTFE) membrane surface for water desalination.^[^ ^27^ ^]^ In this system, SADG is placed at the bottom of a transparent sealed chamber containing salt water, and the vapor escapes from the bottom PTFE membrane without salt precipitation in a long‐term solar desalination. However, this system has a low evaporation efficiency (49%) under one‐sun irradiation (1 kW m^−2^) because solar absorber directly heats bulk seawater, resulting in low heat utilization of the system. Zhu and co‐workers reported a flexible and salt resistant Janus absorber that demonstrated stable water output (1.3 kg m^–2^ h^–1^, over 16 days) under one‐sun, with a solar efficiency of 72%.^[^ ^28^ ^]^ Meanwhile, Chen and co‐workers reported a Janus evaporator with low tortuosity and demonstrated a stable evaporation rate (1.24 kg m^–2^ h^–1^) during continuous solar desalination tests (100 h) in 3.5 wt% NaCl solution under one‐sun.^[^ ^29^ ^]^ The asymmetric wettability designing of Janus evaporator enables the hydrophobic layer out of the water for heat localization and the hydrophilic layer in the water for continuous water pumping. However, the efficiency was much lower than the typical reported evaporation performance (over 80%) of interfacial solar evaporation. Hu and co‐workers reported an artificial channel‐array wood‐based SADG, which can rapidly exchange the salt with the bulk solution, enabling real‐time self‐regeneration of the evaporator with stable evaporation efficiency (≈75%) at wide concentration range salt solution (0–20 wt% NaCl) under one‐sun irradiation.^[^ ^30^ ^]^ However, the millimeter‐sized drilled channels occupy the large area ratio of wood‐based SADG (≈20% of the total insulation area) resulting in the increased heat loss to the bulk solution and mediocre evaporation performance comparing the reported SSG. Hence, to achieve long‐term efficient evaporation performance of SADG, there is still a long way to go. The advanced structure design for self‐regeneration, high heat utilization efficiency, together with fast vapor escaping are comprehensive solutions.

**Figure 1:**
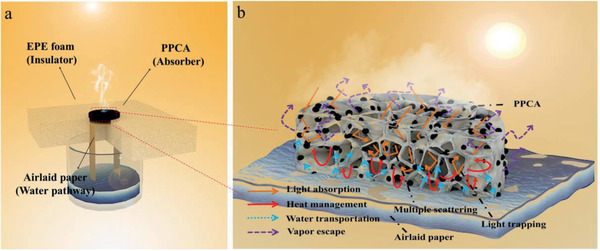
Schematic diagram of a) MTS device and b) PPCA under solar‐assisted desalination experiment.

Here, based on our previous work on mimetic transpiration system (MTS) and frozen photothermal aerogel technology,^[^ ^31^ ^]^ an integrative self‐cleaning chitosan‐based photothermal aerogel for long‐term solar‐assisted desalination is exhibited in **Figure** **1**. 3D honeycombed chitosan (CS)‐based aerogel was prepared using phase transition freeze‐drying method, where amorphous carbon powders (pomelo peel carbonization powders, named as PPCPs) were evenly distributed on porous CS backbone. During solar desalination, the aerogel as photothermal layer was placed on MTS device, in which the air‐laid paper as water pathway supplied water for aerogel, wrapping with expandable polyethylene (EPE) foam to restrict the heat conduction from aerogel to bulk water. Thanks to unique porous structure and super hydrophilic, integrative CS‐based aerogel exhibites self‐heat management, continuous water supply, large vapor escape space, and long‐term salt resistant abilities. Moreover, owing to PPCPs homogeneous intersperse on the hydrophilic CS membrane, the inducing transient salinity difference of fast‐exchange solar driven‐vapor on every PPCPs can be quickly balanced by CS skeleton. Therefore, the integrative CS‐based aerogel performs stable and efficient evaporation performance (efficiency greater than 90%), and superior salt resistance even in high concentration brine (up to 10 wt%), which has broad application prospects in long‐term solar desalination and industrial wastewater treatment.

## 2 Results and Discussion

### 2.1 Structure Design and Characterization of Integrative CS‐Based Aerogel

**Figure 2:**
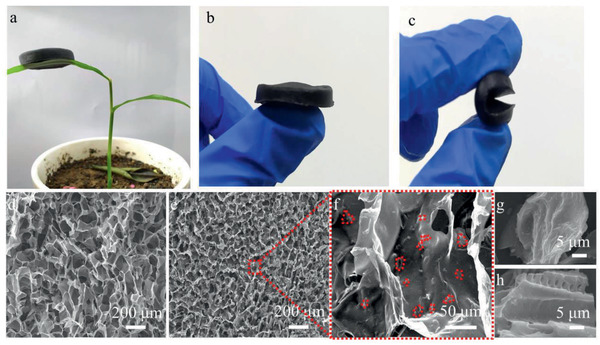
a) Photograph of PPCA loading on plant. b,c) Diagrams of wet aerogel under external loading. d) Image of chitosan aerogel. e,f) Images of PPCA. g,h) Images of PPCP.

For SADG, the structure stability is the precondition of long‐term desalination. Amorphous carbon has broad‐band light absorption and chemical stability, which can be fabricated by biomass carbonization in low‐cost way. CS is a biocompatibility material and can be extracted from crustaceans. The integrative SADG consists of PPCPs and CS aerogel (denoted as PPCA) that can be readily prepared by assembling the PPCPs with CS solution and directly freeze drying. As a proof of concept, PPCPs can be stably loaded on CS chains by electrostatic adsorption (Table S1, Supporting Information). Based on our previous work, the structure and pore size of aerogel can flexibly controlled by adjusting frozen preferential direction and temperature.^[^ ^31^ ^]^ Here, a 3D honeycomb‐like porous PPCA is fabricated by a lyophilizer with circular frozen system, which promotes the growth of ice crystals from outside to inside along the radial direction and then removes the ice template by sublimation. The porous structure of PPCA results in ultralight performance and can stand on the green shoots of ginger (**Figure** **2**a). Moreover, after alkali treatment to remove redundant acetate ion, the aerogel has excellent resilient ability under wetting conditions. The PPCA not only can easy be bent, but also can quickly recover when contacts water, even if after suffering extremely extruded by external forces (Figure 2a,b), which ensures that it can operate normally under harsh conditions. As Figures 2d–f show, the PPCPs uniformly loading on CS pore wall has good compatibility with CS and the original structure of the CS aerogel is preserved. The surface of PPCPs is folds and tubular (Figure 2g,h), which is benefit to light absorption.

It should be noted that *x*, *y* in PPCP*_x_* and PPCA*_y_* stand for different carbonized temperatures of PPCP and their corresponding PPCA, respectively (the abbreviations are shown in Experimental Section of Supporting Information). The chemical compositions of PPCA were conducted on Raman spectra, Fourier transform infrared spectroscopy and X‐ray photoelectron spectroscopy technology to comprehensive analyze (Figure S1, Supporting Information), which demonstrated the PPCA contains plenty of hydrophilic groups (hydroxyl, carboxyl, and amino group),^[^ ^32^, ^33^, ^34^, ^35^ ^]^ and these hydrophilic groups play key roles in water transportation as well as structure springback. Futhermore, the pore character and thermal management ability of PPCA were shown in Figure S2 and Table S2 in the Supporting Information. The PPCA has low thermal conductivity (≈0.035 W m^−1^ K^−1^) and high porosity (≈94%), which is benefit to heat management and vapor escape while solar‐assisted desalination is operated.

### 2.2 Material and Solar Absorption Optimization

The thermal decomposition process of raw pomelo peel can be separated from three stages by thermogravimetric analysis and derivative thermogravimetry device. As shown in Figure S3 in the Supporting Information, the first stage of raw pomelo peel's weight loss is 9.3% from 30 to 197 °C, it can be ascribed to the removal of bound water molecule and some volatile organic compounds decomposition. The second stage is the temperature range from 197 to 305 °C, the weight loss of pomelo peel is 17.4%, which involves undecomposed organic compounds and hemicellulose. The last stage involves hemicellulose, cellulose, and lignin decomposition at the temperature range from 305 to 900 °C.^[^ ^36^ ^]^

**Figure 3:**
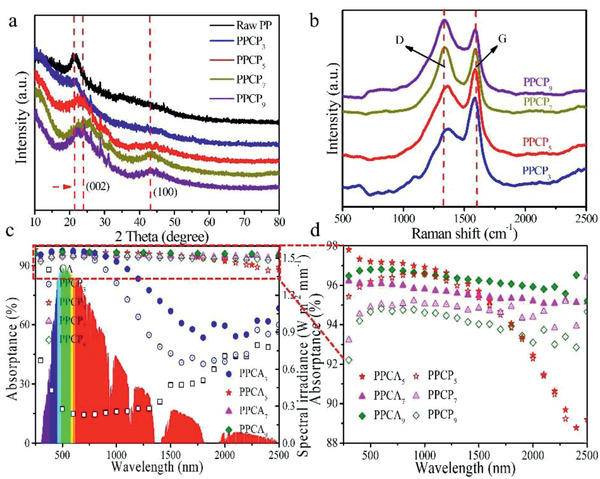
a) XRD spectra of raw PP and PPCP*_x_*. b) Raman spectra of PPCP*_x_*. c,d) Absorptance spectra of chitosan aerogel (CA), PPCP*_x_*, and PPCA*_y_* at the range of 250–2500 nm.

To investigate the relationship of light absorption performance with disorder of amorphous carbon, the PPCPs with different carbonization temperatures were characterized. In **Figure** **3**a, XRD spectra of different PPCP samples shows that the characteristic peak width of (002) lattice plane increased with the increase of pyrolysis temperature. Moreover, when the pyrolysis temperature reaches more than 700 °C, another characteristic broad peak appears at the (100) lattice plane.^[^ ^37^ ^]^ The appearance of broadened graphite diffraction peak and new diffraction peak indicates that PPCPs are amorphous carbon structure, which is the result of the structure fracture of graphite carbon during pyrolysis. The characteristic peaks of stretching vibration of homonuclear diatomics in PPCP samples were detected by Raman spectroscopy. As shown in Figure 3b, there are two characteristic peaks located around 1343 and 1587 cm^−1^, which are related to the D and G bands, respectively. As for PPCP samples, the intensity of D band increased, indicating the carbon‐based lattice defects increased while the pyrolysis temperature rising. As shown in Figures 3c,d, PPCA samples have extensive full‐spectrum absorption at the range of250–2500 nm, which demonstrates that CS has a good compatibility with PPCPs. Moreover, compared with PPCP samples, the light absorption of all PPCA samples were increased becausethe unique porous structure of aerogel have light multiple‐scattering and light traps enhancement effects. PPCA_3_ has several absorption attenuation peaks at the near infrared region, which is due to partially undecomposed organic functional groups remains in PPCP_3_. The PPCA_5_ has the best light absorption performance of 96% at full‐spectrum (The absorptance calculation process of sample was shown in the Supporting Information).

### 2.3 Solar Steam Generation Optimization Experiment

Based on the excellent optical properties, superhydrophilicity and good thermal management ability of PPCA samples, it is a good candidate material for solar photothermal conversion. In this work, PPCA samples were loaded on MTS device to conduct solar steam generation experiment (Figure 1a). Basically, the PPCA sample was embedded in the EPE foam as planar system. As shown in Figure S4a in the Supporting Information, when the illumination time reached 10 min, the mass loss rate of each sample tended to be stable, indicating that aerogels had fast photothermal conversion abilities. The evaporation rate (0.91 kg m^−2^ h^−1^) of CA is faster than pure water (0.6 kg m^−2^ h^−1^). As for the planar system, the PPCA_5_ has evaporation rate of 1.57 kg m^−2^ h^−1^ and evaporation efficiency of 97.1% (Figure S4a, Supporting Information), which is better than conventional planar SSG.^[^ ^2^, ^6^, ^8^, ^10^ ^]^ The MTS has heat utilization efficiency of 90%. It demonstrated that the PPCA‐MTS has excellent solar steam generation performance. More detail information of photothermal conversion efficiency and thermal utilization of PPCA samples and MTS were discussed in calculation section in the Supporting Information.

Moreover, in the MTS, when PPCA samples are placed on EPE foam as open system (Figure S4b, Supporting Information), the mass loss rates of each sample are faster compared with the planar system under the same experimental conditions. It is attributed to the unique open 3D honeycomb structure of the integrated aerogel that provides more steam escape channels than the planar system. The PPCA_5_ sample with open system has the best evaporation rate of 1.78 kg m^−2^ h^−1^, which breaks the limit rate (1.47 kg m^−2^ h^−1^) of the interface system.^[^ ^15^, ^38^, ^39^ ^]^ The increased evaporation rate of open system can be attributed to its good heat management ability and the additional energy gain form ambient environment, which increased the vapor production.^[^ ^38^, ^39^, ^40^ ^]^ The evaporation rate of PPCA_5_ can be further improved by rational designing system structure, each PPCA_5_ as a building block unit can be simply accumulated to enlarge lateral area. As the lateral areas of PPCA_5_ increased (Figure S5, Supporting Information), it realizes the utilization of the environmental energy.^[^ ^38^ ^]^ Specifically, the enhancing solar steam generation performance of 2.04 kg m^−2^ h^−1^ was obtained.

### 2.4 Long‐Term Solar‐Assisted Desalination Experiment

**Figure 4:**
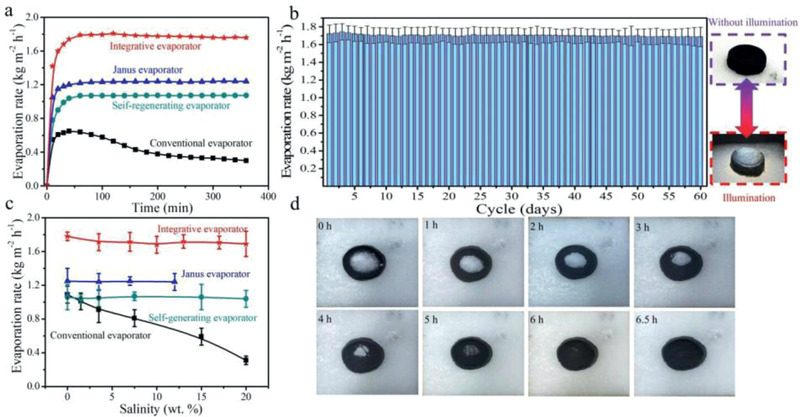
a) Comparison of long‐term solar‐assisted desalination performance between integrative evaporator and other evaporators in 3.5 wt% brine. b) The evaporation rate of integrative evaporator during a static‐continuous illumination experiment in 3.5 wt% brine for 60 days. c) Comparison of the evaporation capacity of integrative evaporator and other evaporators with different concentrations of brine. d) Self‐cleaning experiment of an integrated evaporator in 3.5 wt% brine.

To evaluate the solar‐assisted desalination performance of integrative aerogel, we carefully studied the long‐term evaporation rate of the PPCA_5_ open system (Integrative evaporator) with one unit on the MTS device in 3.5 wt% NaCl (average salinity of seawater). As shown in **Figure** **4**a, the integrative evaporator fast reached its highest evaporation rate after 1 h under 1 kW m^−2^ solar irradiation. And the integrative evaporator has stable evaporation performance (1.70 kg m^−2^ h^−1^) in the sequence 6 h test, which is much higher than Janus evaporator and self‐regenerating evaporator.^[^ ^29^, ^30^ ^]^ Moreover, there was no visible salt on the surface of evaporator. The stable solar‐assisted desalination performance of integrative evaporator was evaluated from a static‐continuous illumination experiment by soaking the aerogel in 3.5 wt% NaCl solution for 60 days (Figure 4b). The evaporator was installed on MTS to conduct solar‐assisted desalination, which was continuously irradiated for 6 h twice daily. During the experimental interval, the sample was still kept in 3.5 wt% NaCl solution. The evaporator showed a stable evaporation rate of ≈1.70 kg m^−2^ h^−1^ under one‐sun and without structure deterioration last for 60 days. It demonstrates that the integrative evaporator has excellent cycle ability in solar‐assisted desalination. The stability and evaporation performance of integrative evaporator was also compared to current work in Table S7 in the Supporting Information.

The solar‐assisted desalination properties of integrative evaporator were evaluated by a series of NaCl solutions with different concentrations ranging from 0 to 20 wt%. As shown in Figure 4c, the evaporation rates of the integrative aerogel are higher than Janus evaporator and self‐regenerating evaporator in all the concentrations tests. Moreover, we carefully conducted long‐term solar‐assisted desalination experiments of the integrative evaporator with different concentration salt solutions to investigate the stability of steam performance, which demonstrated that the integrative evaporator had stable evaporation performance of salt concentration solutions ranging from 0 to 10 wt%. When the salt concentration is more than 10 wt%, the performance of integrative aerogel is gradually decreased. The time of salt precipitation is corresponding to the change point of evaporation rate, which is used to evaluate salt tolerance ability of the integrative evaporator to different salt concentration solutions. As shown in Figure S6 in the Supporting Information, the integrated evaporator has no salt precipitation under the salinity condition of 3.5 wt%, even continuous illumination lasts to 12 h. Under conditions of 10 and 20 wt%, the salt precipitation of integrative evaporator occurred at 8.2 and 1.9 h, respectively. It is proved that the integrative evaporator performs well in saline solutions of 1–10 wt%.

The salt tolerance mechanism of integrative evaporator is similar to mangrove forest. The cell of mangrove forest has high osmotic pressure that allows it to survive near the sea. As for integrative evaporator (Figure S7, Supporting Information), the interconnected CS chain is superhydrophilic and can quickly absorb water and reserve it. The PPCP evenly distributes on the CS chains. When the integrative evaporator is conducted on desalination experiment, the rapid gas‐liquid exchange induces local salt concentration increase on the PPCP. It achieves real‐time salt exchange between PPCP and CS chains under the competition of evaporation and temperature difference driving force, as a result, the locally salinity gradient of the evaporator can be quickly balanced. Specifically, the spatial distribution of photothermal material on aerogel skeleton has a great influence on the evaporation performance and stability of evaporator, which was demonstrated by seawater desalination experiments with inhomogeneous aerogels (Figure S8, Supporting Information). Based on the real‐time salt exchange, the integrative evaporator exhibits high efficient evaporation performance for seawater. CS chains play the key role of water supply as well as balancing the salt concentration in the evaporator, which extends the time to reach the saturation concentration of salt for the evaporator. However, when the concentration of brine treated by the integrated evaporator exceeds 10 wt%, this efficient and stable desalination performance will be weakened. Compared with natural seawater, the shrink of controllable real‐time salt exchange ability of CS chains in high‐concentration brine makes the evaporator reach salt saturation earlier at high speed evaporation rate under solar‐assisted desalination experiment (Figure S9, Supporting Information). Moreover, the 1 g NaCl crystal was placed on the top of the integrated evaporator. As shown in Figure 4d, after 6.5 h natural evaporation, the salt at the surface of evaporator was disappeared, which demonstrated that the integrative evaporator has extremely salt tolerance and self‐cleanable abilities in seawater.

**Figure 5:**
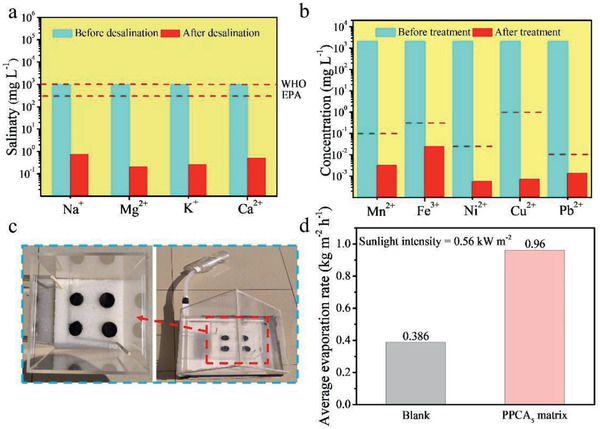
a) The salinities of different mineral elements before and after desalination by integrative evaporator. b) The concentrations of different heavy metal ions before and after water treatment by integrative evaporator. c) Photograph of self‐made outdoor desalination device. d) Average evaporation rate of blank and PPCA_5_ matrix evaporator.

For investaging mineral elements content in the collected condensate water, the simulated desalination performance of integrated evaporator was tested. The result shows that the mineral elements of the collected water are much lower than the World Health Organization (WHO) and the US Environmental Protection Agency (EPA) standards for drinkable water (**Figure** **5**a).^[^ ^41^ ^]^ Furthermore, to demonstrate treatment ability of the multiple pollution water, a simulated industrial wastewater experiment of the integrative evaporator was also conducted. After the treatment (Figure 5b), all of the concentrations of the heavy metal ions are below of the government standard for the heavy metal ions in the drinking water (GB5749‐2006).^[^ ^8^ ^]^

A proof‐of‐concept prototype consisted of 2 × 2 matrix SADG using the PPCA_5_ as the building block, which was placed in a polymethyl methacrylate cover (Figure 5c). The 3.5 wt% brine at the bottom of the container was used as the simulated seawater and the condensed vapor during the outdoor experiment would be collected in a bottle. The experiment was carried out from 10:00 to 16:00 under natural sunlight with a solar flux of about 0.56 kW m^−2^. The average evaporation rate of the SADG with PPCA_5_ was calculated to 0.96 kg m^−2^ h^−1^ (Figure 5d), which is much higher than that of the blank SADG under the same illumination condition. The SADG with PPCA_5_ shows a capacity to produce about 11.52 kg m^−2^ d^−1^ of drinkable water under long‐term desalination with natrual sunlight. It opens up a promsing path for sustainable, low‐cost and high‐performance seawater desalination.

## 3 Conclusion

An integrated evaporator with high absorption, thermal management, water transportation and salt tolerance abilities is prepared through structural and material optimization. The evaporator consists of renewable amorphous carbon and CS chains, which is environmental‐friendly and structure stable for long‐term solar desalination. The advanced structural design endows the evaporator with integrative properties, enabling long‐term desalination in 3.5 wt% NaCl solution under one‐sun with greater than 90% efficiency, which is better than the reported evaporators. Moreover, owing to the real‐time salt exchange between PPCP and CS chains of the evaporator, which can perform stable steam generation without salt precipitation even in highly brine water (up to 10 wt%). The integrated evaporator exhibits excellent solar‐assisted desalination performance, high salt tolerance of integrative evaporator in brine water, and superior heavy metal removal ability, indicating a promising prospect for solar‐assisted desalination and other industrial wastewater treatment.

## Conflict of Interest

The authors declare no conflict of interest.

## Supporting information

Supporting Information
