# Supplementary material for: Self‐Cleaning Integrative Aerogel for Stable Solar‐Assisted Desalination
Source: Glob Chall. 2020 Dec 16;5(1):2000063. doi: 10.1002/gch2.202000063 (PMC7788587; doi:10.1002/gch2.202000063)
Supplement: Supplementary file 1 — Supporting Information [file GCH2-5-2000063-s001.pdf]

© 2020 Wiley-VCH GmbH

Global Challenges
Open Access

Supporting Information

for *Global Challenges*, DOI: 10.1002/gch2.202000063

Self-Cleaning Integrative Aerogel for Stable Solar-Assisted Desalination

Yufei Gu, Xiaojiang Mu, Pengfei Wang, Xiaoyang Wang, Yongzhi Tian, Anyun Wei, Jiahong Zhang, Yulian Chen, Zhiqiang Sun, Jianhua Zhou, and Lei Miao**

WILEY-VCH

© 2020 Wiley-VCH GmbH

Supporting Information

Self-cleaning Integrative Aerogel for Stable Solar-assisted Desalination

Yufei Gu, Xiaojiang Mu, Pengfei Wang, Xiaoyang Wang, Yongzhi Tian, Anyun Wei, Jiahong Zhang, Yulian Chen, Zhiqiang Sun, Jianhua Zhou, Lei Miao**

Experimental Section

Preparation of $PPCP_x$: Pomelo peel (recycled from fruit shop in Guilin) was suffered simple cleaning treatment, and then was dried in oven (DHG-9075A Qixin Scientific Instrument Co. LTD Shanghai) at 80 ºC. Next, the drying pomelo peel was carbonized in a vacuum tube furnace (OTF-1200X, Kejing Materials Technology Co., Ltd. Hefei) under nitrogen atmosphere with a flow rate of 20 sccm, by adjusting different carbonization temperature for 300, 500, 700, and 900 ºC, respectively. Final, each carbonized product was grinded and sieved through a stainless steel sieve of 120, 300, and 500 meshes to fabricate different pomelo peel carbonization powder ($PPCP_x$, *x* can be 3, 5, 7, and 9, which refers to different carbonization temperature).

Preparation of $PPCA_y$: Each 0.1 g $PPCP_x$ with the size of 500 mesh was dispersed in 6 mL CS solution, and then, freeze drying by a lyophilizer (ALPHA 2-4 LSC, CHRIST, Germany) to fabricate $PPCA_y$ (*y* can be 3, 5, 7, and 9, which refers to different carbonization temperature).

Preparation of $PPCA_5$-Z: Each 0.1 g $PPCP_5$ with the different sizes (120, 300, 500 mesh) was dispersed in 6 mL CS solution, and then, freeze drying by a lyophilizer to fabricate $PPCA_5$-Z (*Z* can be 120, 300, 500).

Characterization: The pyrolysis process of raw PP was investigated by thermogravimetric analysis and differential thermal analysis device (TGA-DTA, SDT Q600, TA Instruments, USA), with ramp 5 ºC min^{-1} to 900 ºC, in nitrogen atmosphere. The morphology and structure of raw PP, $PPCP_x$, and $PPCA_y$ samples were investigated by a scanning electron microscope (SEM, S-4800, Hitachi, Japan). X-ray diffraction (XRD, D8-ADVANCE, Bruker, Germany) with Cu Kα radiation was conducted on phase analysis. The chemical component of materials was comprehensive analysis by Raman spectroscopy (LabRAM HR, Horiba, French) with objective X 50, grating 1800 gr nm^{-1}, laser 532 nm, Fourier transform infrared spectrometer (FTIR, Nicolet IS10, Thermo Fisher, USA) with a KBr disc in the range of 400-4000 cm^{-1}, X-ray photoelectron spectroscopy (XPS, ESCALAB 250Xi, Thermo Fisher, USA) with an Al K-

alpha anode emitter as the excitation source. The absorptance spectrum of each sample was transformed from its diffuse reflectance spectral and transmittance spectral, which measured from 2500 to 250 nm via a UV-Vis-NIR spectrometer (V-570, JASCO, Japan) equipped with an integrating sphere. The infrared reflectance spectral of materials were measured by a FTIR spectrometer (Nicolet IS10, Thermo Fisher, USA) connecting an IntegratIR^{TM} mid-infrared integrating sphere with Mercury-Cadmium-Telluride (MCT) detector (PIKE). The macropore parameter of $PPCA_y$ was measured by mercury porosimetry (AutoPore IV 9500, Micrometritics, USA). Thermal conductivity of $PPCA_y$ was measured by thermal conductivity instrument (TCi, C-therm, Canada). Zeta potentials of materials were investigated by laser particle analyzer (MS2000, Malvern, UK).

Solar steam generation experiment: The whole process of the experiment was conducted at an ambient temperature of 25 ± 2 °C and a relative humidity of 48 ± 2%. The pretreatment $PPCA_y$ sample was loaded on homemade MTS to conduct experiment. A xenon lamp (CEL-S500/350, ZJJY, Beijing) with an AM1.5 optical filter was used as the light source. A piece of Fresnel lens with 20 cm focal length was applied to enhance incident light. During SSG experiment, light intensity of 1 kW m^{-2} was calibrated by an optical power densitometer (843-R, Newport, USA) with a thermopile sensor (919P-010-16, Newport, USA). The mass change of sample was recording by a high-precision electric balance (ATX224, Shimadzu, Japan) for 60 min at constant condition. The surface temperature distribution of $PPCA_y$ was captured by an infrared camera (E60, FLIR, USA). The temperature of vapor was measured by a thermal sensor probe (BD-PT100-3022A).

In long-term solar-assisted desalination experiments, brine of different concentrations (3.5, 7, 10, 13, 17 and 20 wt%) was prepared by dissolving NaCl in water in each proportion. During laboratory solar wastewater treatment, $MnSO_4{\cdot}H_2O$, $Fe(NO_3)_3{\cdot}9H_2O$, $(CH_3COO)_2Ni{\cdot}4H_2O$, $CuCl_2{\cdot}2H_2O$, $(CH_3COO)_2Pb$ were selected as model heavy metal ion (Mn^{2+}, Fe^{3+}, Ni^{2+}, Cu^{2+}, Pb^{2+}) contamination to evaluate solar-assisted water treatment performance. The

concentration of the heavy metal in the production fresh water was measured by inductively coupled plasma atomic emission spectroscopy (ICP-AES, Optima 8000, PerkinElmer, Waltham, MA, USA). Moreover, NaCl, $MgCl_2$, KCl, and $CaCl_2$ were selected for simulated desalination experiment, the production water was measured by ICP-AES for evaluating water quality.

The outdoor experiment was performed through a prototype made of polymethyl methacrylate (PMMA) box with double inclined planes, and polyvinyl chloride tube as the water chute for collecting the condensed water. An expanded polystyrene foam with 2×2 array of $PPCA_5$ with an effective area of 28 cm^2. The simulated seawater containing 3.5 wt% of NaCl was placed into the bottom of the prototype. The outdoor experiment was carried out under natural sunlight with a solar flux of ~0.56 kw m^{-2}.

Calculation

Absorptivity calculation:

According to Kirchhoff's law, the absorptivity was calculated by Eq. (1):

$$A = 1 - R - T \tag{1}$$

Where *R* and *T* are the reflectivity and transmissivity, respectively. The absorptivity values of different samples were calculated based on Eq. (2), resulting from the solar energy dependent on the wavelength in the solar spectrum.[1]

$$\alpha = \frac{\int_{250}^{2500}[1-\mathrm{R}(\lambda)-\mathrm{T}(\lambda)]P_{sun}(\lambda)\mathrm{d}\lambda}{\int_{250}^{2500}P_{sun}(\lambda)\mathrm{d}\lambda} \tag{2}$$

Where, $P_{sun}(\lambda)$ is the normal solar irradiance defined by the ISO standard 9845-1 (1992) for air mass (AM)1.5. The obtained absorptivity values of samples are shown in Tale S3.

Evaporation rate calculation:

The evaporation rate is a key factor to evaluate the photothermal conversion performance of SSG. The evaporation rate *v* is given by

$$v = \frac{\dot{\mathrm{m}}}{A_{proj}} \tag{3}$$

Where $\dot{m}$ is the mass flux of loss water under SSG, which can be represented as the slope of the mass loss curve, obtained by linear fitting in the steady state. A_{proj} is the projection area of sample.

Moreover, the mass flux of loss water generated by the sample consists of natural evaporation($\dot{m}_{nature}$) and light-driven evaporation($\dot{m}_{light}$). Which is given by

$$\dot{\mathrm{m}} = \dot{\mathrm{m}}_{natrue} + \dot{\mathrm{m}}_{light} \tag{4}$$

Photothermal conversion efficiency calculation:

Based on the unique of system, the photothermal conversion efficiency η_1 can be represented as follow:

$$\eta_1 = \frac{\dot{\mathrm{m}}_{light}(C_p \times \Delta T + \Delta H_{vap})}{c_{opt} I A_{proj}} \tag{5}$$

Specifically

$$\Delta H_{vap} = RT_c\left(A\tau^{\frac{1}{3}} + B\tau^{\frac{2}{3}} + C\tau + D\tau^2 + E\tau^6\right) with\, \tau = 1 - \frac{T_{vap}}{T_c} \quad (6)$$

Where c_{opt} is the optical concentration, I is the nominal direct solar illumination (1 kW m^{-2}). $C_p \times \Delta T$ is sensible heat. C_p is the specific heat capacity of water (4.18 J g^{-1} K^{-1}). ΔT represents the difference between the vapor temperature and the ambient temperature. ΔH_{vap} is latent heat, which depends on Eq. (6). Where T_{vap} represents vapor temperature. *A, B, C, D, E,* τ, T_c are standard constant.[2-3] The photothermal conversion efficiency of each sample is shown in Table S4.

Emissivity calculation:

The thermal emissivity refers to the proportion of blackbody radiation absorbed by the sample to the total black body radiation, which is dependent on the nature and surface state of the materials. The emissivity ε of the material can be obtained by measuring its infrared reflectance spectrum and calculating, the formula is as follows:[1]

$$\varepsilon = \frac{\int_{25\mu m}^{2.5\mu m}[1-R(\lambda)]P_B(\lambda)d\lambda}{\int_{25\mu m}^{2.5\mu m}P_B(\lambda)d\lambda} \quad (7)$$

Where $P_B(\lambda)$ is the spectral radiance of blackbody at a temperature T. According to Plank's law:

$$P_B(\lambda) = \frac{C_1}{\lambda^5[e^{C_2/\lambda T}-1]} \quad (8)$$

Where C_1 and C_2 are constant, $C_1 = 3.743 \times 10^{-16}\ W\ m^{-2}$, $C_2 = 1.4387 \times 10^{-2}\ m\ K$. The measurement temperature $T = 25$ °C. The reflectance spectra of samples and temperature were put into the formula (3) and (4) to perform the calculation by a Matlab program.[4] The obtained emissivity values of samples are shown in Tale S5.

Energy loss calculation:

For planar systems, there are four paths of energy loss: surface reflection, heat radiation, heat convection and heat conduction.

Specifically, the SSG experiment was conducted in lab environment. The temperature was adjusted at 25 ± 2 °C, and the illumination intensity was controlled at 1 kW m^{-2} by light source.

The surface reflection happens on the surface of aerogel, P_{ref} is given by

$$P_{ref} = (1-\alpha)P_{light} \tag{9}$$

Where $P_{light} = l\ kW\ m^{-2}$.

The heat radiation ($P_{ra,\ vap}$) between system and vapor is calculated by the Stefan–Boltzmann equation

$$P_{ra,vap} = \sigma \times \varepsilon \times A_{proj} \times (T_{ab}^4 - T_{vap}^4) \tag{10}$$

Where $\sigma=5.67\times 10^{-8}\ W\ m^{-2}\ k^{-4}$ denotes the Stefan–Boltzmann constant. T_{ab} is the temperature of absorber.[1]

The heat convection ($P_{cv,\ vap}$) between system and vapor is defined by Newton's law of cooling

$$P_{cv,vap} = h \times A_{vap} \times \left(T_{ab} - T_{vap}\right) \tag{11}$$

Where $h = 5\ W\ m^{-2}\ k^{-1}$ denotes the convective heat transfer coefficient of the planar system.[1]

The heat conduction mainly consists of heat transferring from system to water and EPE foam. The heat conduction from system to water ($P_{cd,\ water}$) is given by

$$P_{cd,water} = C_p m_1 \Delta T_1 \tag{12}$$

where m_1 is the mass of the remaining bulk water, and ΔT_1 is the temperature change of bulk water.[1]

The heat conduction from system to EPE ($P_{cd,\ EPE}$) is given by

$$P_{cd,EPE} = \kappa \times A_{transfer} \times \frac{\Delta T_2}{\Delta x} \tag{13}$$

Where κ is the thermal conductivity of EPE foam. $A_{transfer}$ is the heat transfer area of the absorber towards EPE foam, and $\frac{\Delta T_2}{\Delta x}$ is the temperature gradient of the absorber towards EPE

foam.[1]

The thermal utilization η_2 of each sample with MTS device is given by

$$\eta_2 = 1 - \frac{P_{ref} + P_{ra,vap} + P_{cv,vap} + P_{cd,water} + P_{cd,EPE}}{P_{light}} \quad (14)$$

The thermal utilization and every step of heat loss of sample are listed in Table S6. And the SSG energy flow and resistance network diagram of $PPCA_5$ was shown in Figure S10.

Figures

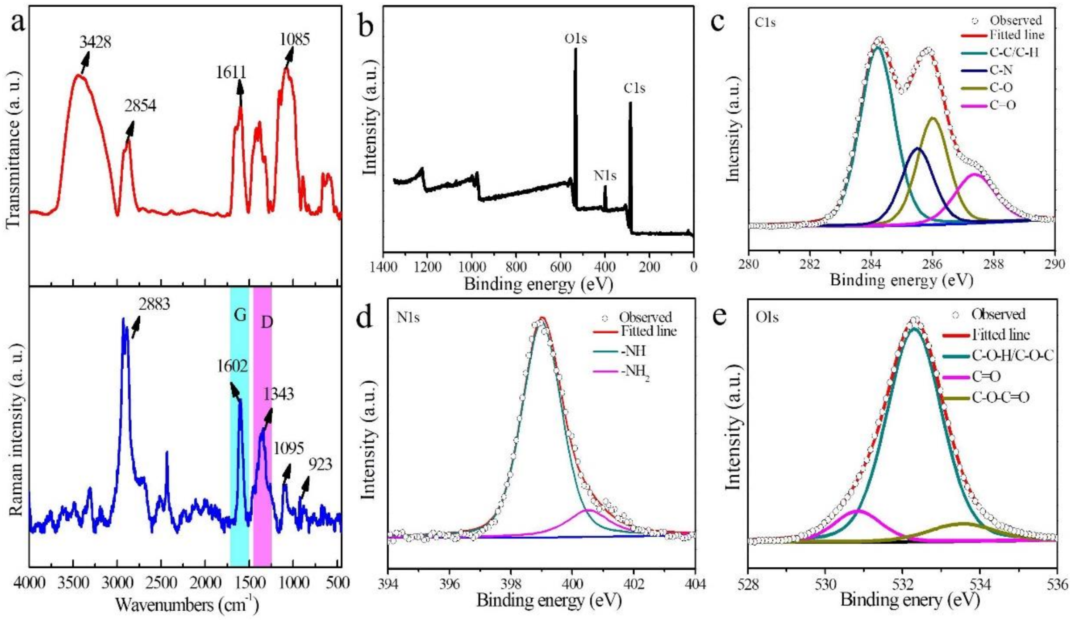

Figure S1. (a) FTIR and Raman spectrum of PPCA. XPS spectral of PPCA. (b) Full spectrum scanning; (c) C1s scan spectrum; (d) N1s scan spectrum; (de O1s scan spectrum.

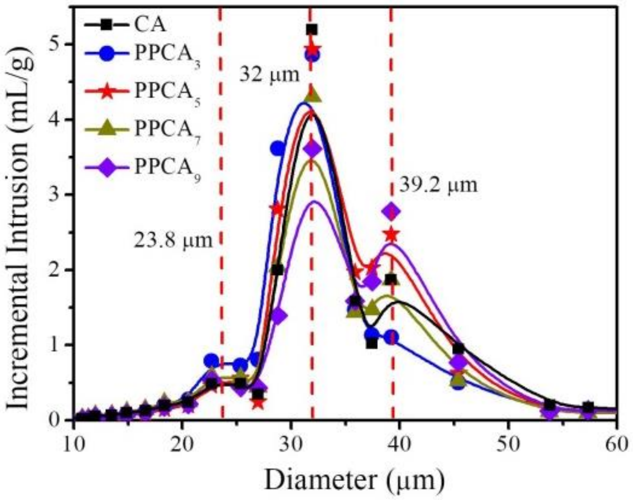

Figure S2. The pore size distribution of each sample by Mercury intrusion porosimetry.

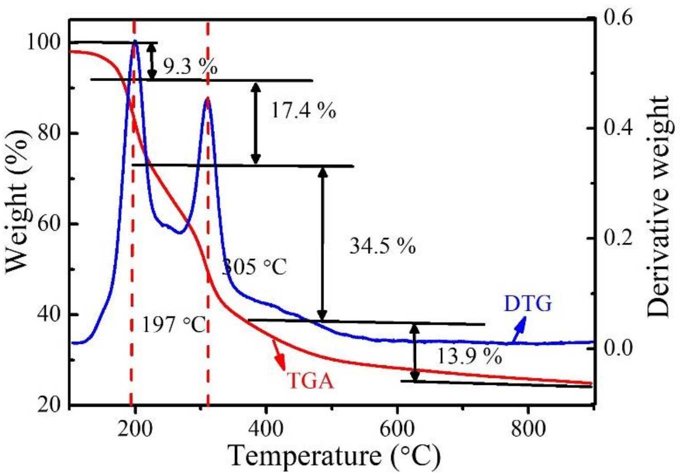

Figure S3. The TGA-DTG analysis of raw pomelo peels.

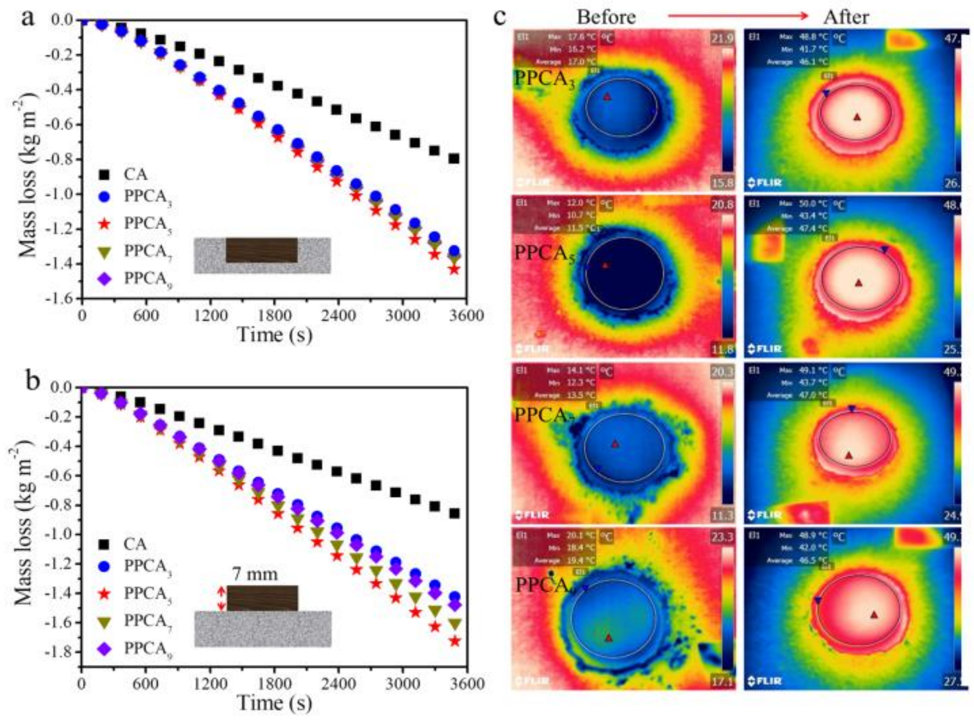

Figure S4 (a), (b) is the SSG mass loss curves of CA and $PPCA_y$ when embedded (planar system) and highlighted (open system), respectively. (c) Infrared images of $PPCA_y$.

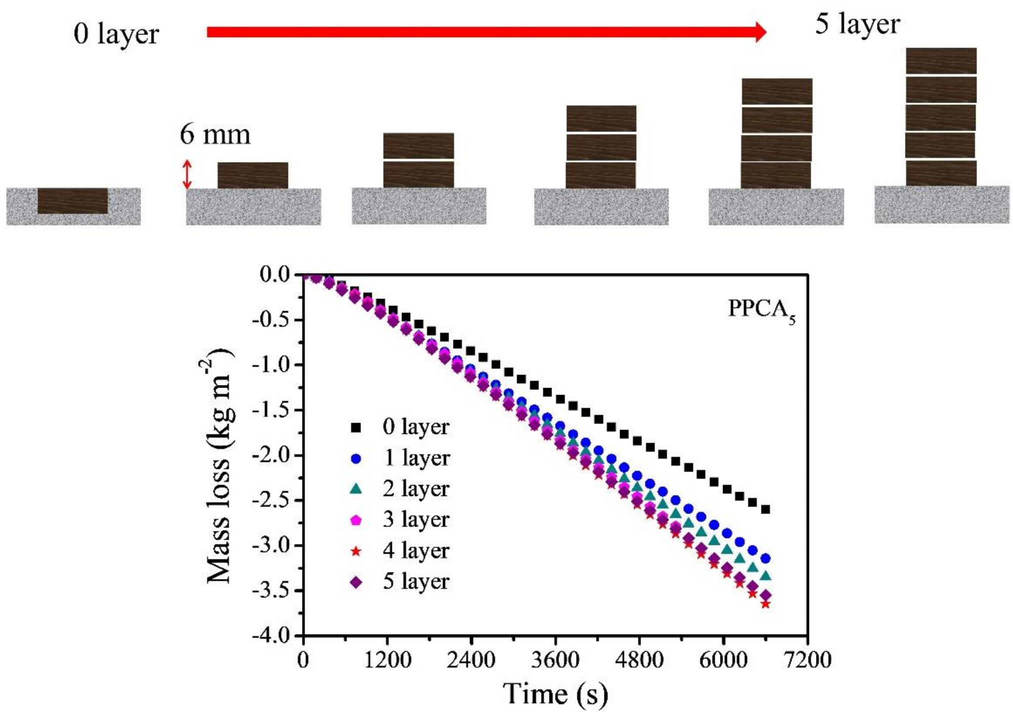

Figure S5. The SSG curves of $PPCA_5$ at different heights.

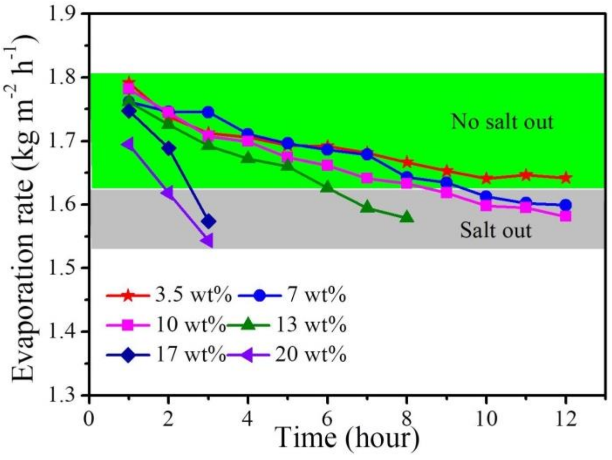

Figure S6. Long-term solar-assisted desalination experiment of integrative evaporator with salt solution of 0-20 wt%.

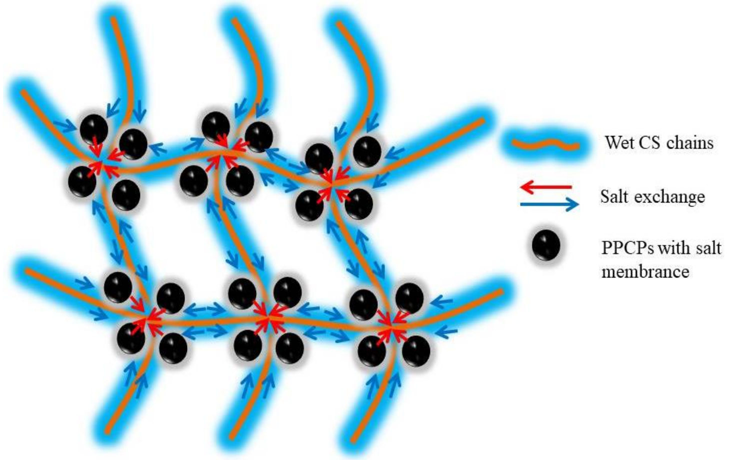

Figure S7. Schematic diagram of aerogel network regulating salt concentration balance.

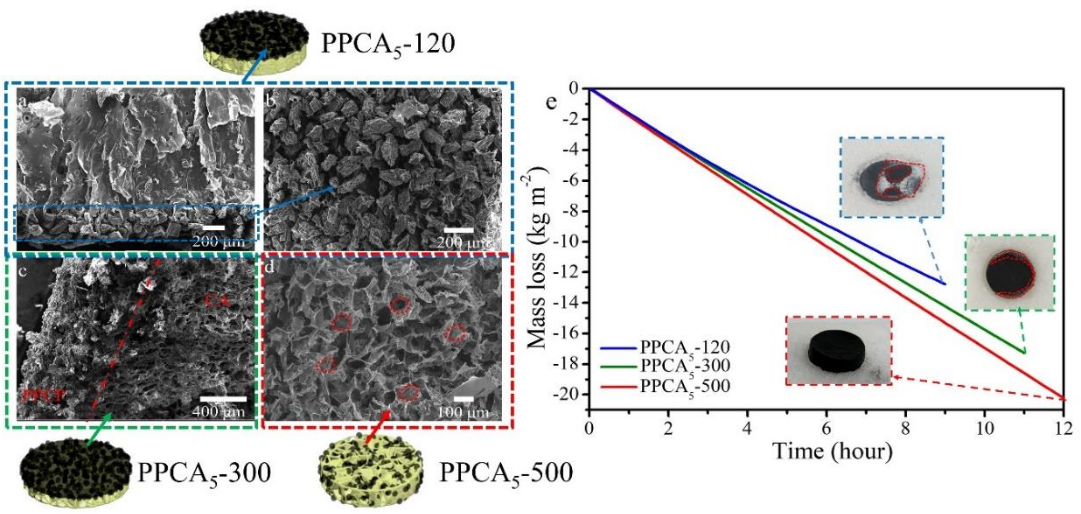

Figure S8. (a-d) The spatial distribution diagrams of PPCP on CA framework. (a), (b) PPCA$_5$-120. (c) PPCA$_5$-300. (d) PPCA$_5$-500. (e) Long-term solar-assisted desalination experiment of each PPCA$_5$-Z.

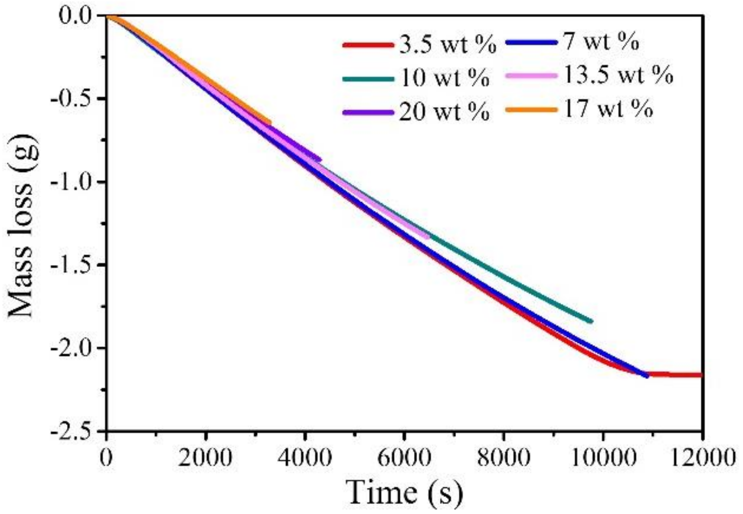

Figure S9. Limit evaporation experiment of integrated evaporator with different salt concentrations.

When integrative evaporator was conducted solar-assisted desalination experiment. With the increase of brine concentration, the point of salting-out shows blue shift.

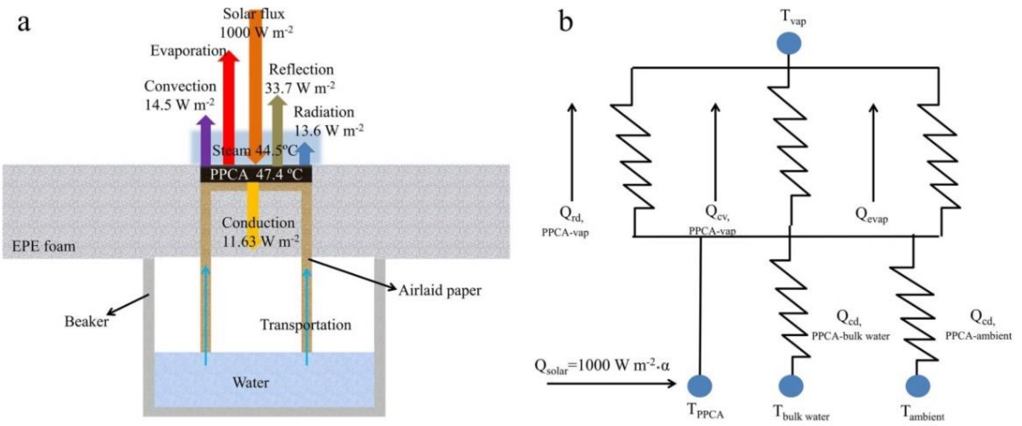

Figure S10 (a), (b) is SSG energy flow and thermal resistance network diagram of $PPCA_5$ embedded in the MTS device, respectively.

Tables

Table S1. The Zeta potential of each component.

Sample	Zeta potential [mV]	Conductivity [mS cm^{-1}]
Water	+3.17	0.0134
$PPCP_3$	-59.4	0.0792
$PPCP_5$	-39.4	0.0664
$PPCP_7$	-29.6	0.304
$PPCP_9$	-24.3	0.29
$CS_{1.5}$	+57.2	5.05
$PPCP_3$/ $CS_{1.5}$	+46.3	0.765
$PPCP_5$/ $CS_{1.5}$	+50.1	0.589
$PPCP_7$/ $CS_{1.5}$	+45.3	0.764
$PPCP_9$/ $CS_{1.5}$	+46.7	0.781

Table S2. Physical properties of each sample.

Sample	Pore diameter [μm]	Porosity [%]	Thermal conductivity [W m^{-1} K^{-1}]
CA	32.9	96.1	0.033
$PPCA_3$	33.3	94.7	0.037
$PPCA_5$	32.7	94.3	0.035
$PPCA_7$	34.6	94.8	0.036
$PPCA_9$	36.4	95.5	0.037

Table S3. The absorptivity of each sample.

Sample	Absorptivity[%]	Sample	Absorptivity[%]
$PPCP_3$	85.42	$PPCA_3$	91.29
$PPCA_5$	96.06	$PPCA_5$	96.80
$PPCA_7$	94.92	$PPCA_7$	95.93
$PPCA_9$	94.53	$PPCA_9$	95.60
-	-	CA	18.96

Table S4 Photothermal conversion efficiency of each sample.

Sample	Evaporation rate [kg m^{-2} h^{-1}]	Natural evaporation rate [kg m^{-2} h^{-1}]	Vapor temperature [°C]	Sensible heat [kJ kg^{-1}]	Latent heat [kJ kg^{-1}]	Efficiency [%]
CA	0.91	0.139	33.5	35.95	2421.76	52.6
$PPCA_3$	1.44	0.151	43.3	78.17	2399.09	88.7
$PPCA_5$	1.57	0.159	44.5	81.93	2396.28	97.1
$PPCA_7$	1.53	0.140	43.8	80.26	2397.92	95.7
$PPCA_9$	1.47	0.110	43.6	79.42	2398.39	93.6

Table S5. The emissivity of each sample.

Sample	Emissivity
CA	0.731
$PPCA_3$	0.709
$PPCA_5$	0.678
$PPCA_7$	0.668
$PPCA_9$	0.667

Table S6 The thermal utilization of each sample.

Sample	Reflection [W m^{-2}]	Radiation [W m^{-2}]	Convection [W m^{-2}]	Thermal conduction [W m^{-2}]	Thermal utilization [%]
CA	405.3	17.8	19	22.09	53.58
$PPCA_3$	45.3	14.3	14	21.75	90.46
$PPCA_5$	33.7	13.6	14.5	11.63	92.66
$PPCA_7$	34.8	15.8	16	15.12	91.82
$PPCA_9$	40.8	14.8	14.5	16.98	91.29

Table S7. The statistic of solar steam generation performance of currently evaporators in brine under one-sun.

Evaporator	Salinity of the water [wt%]	Evaporation rate [kg m^{-2} h^{-1}]	Durability [h]	Reference
Carbonized wood	3.5	1.27-1.38	10	[5]
SiO_2/CNF/CNT	3.5	1.24	100	[6]
MXene/rGO	3.5	1.37	3	[7]
rGO-MWCNT	3.5	1.19	1.67	[8]
CB/PMMA/PAN	20	1.3	0.75	[9]
PTH	3.5	1.15	1	[10]
$PMoS_2$-CC	3.4	1.3	0.5	[11]
TiO_x-stainless steel	0	0.8	8.5	[12]
Graphene foam	2.75	1.4	1.5	[13]
pDA-rGO/PTFE	4	0.72	1	[14]
Nylon–C cloth	3.5	~1.24	2	[15]
Wood/CNT	3.6	1.46	6	[16]
GO/PVA EFMs	3.5	1.34	1	[17]
Carbon/chitosan aerogel (PPCA)	3.5	1.70	12 (Interval test 60 days)	This work

Reference

[1] J. Zhou, Y. Gu, P. Liu, P. Wang, L. Miao, J. Liu, A. Wei, X. Mu, J. Li, J. Zhu, *Adv. Funct. Mater.* **2019**, 29, 1903255.

[2] X. Li, W. Xu, M. Tang, L. Zhou, B. Zhu, S. Zhu, J. Zhu, *P. Natl. A. Sci. India. B* **2016**, 113, 13953.

[3] G. Ni, G. Li, Svetlana V. Boriskina, H. Li, W. Yang, T. Zhang, G. Chen, *Nat. Energy* **2016**, 1, 16126.

[4] X. D. Xiao, L. Miao, G. Xu, L. M. Lu, Z. M. Su, N. Wang, S. Tanemura, *Appl. Surf. Sci.* **2011**, 257, 10729.

[5] P. Liu, L. Miao, Z. Deng, J. Zhou, H. Su, L. Sun, S. Tanemura, W. Cao, F. Jiang, L. Zhao, *Mater. Today Energy* **2018**, 8, 166.

[6] R. Hu, J. Zhang, Y. Kuang, K. Wang, X. Cai, Z. Fang, W. Huang, G. Chen, Z. Wang, *J. Mater. Chem. A* **2019**, 7, 15333.

[7] K. Li, T. H. Chang, Z. Li, H. Yang, F. Fu, T. Li, J. S. Ho, P. Chen, *Adv. Energy Mater.* **2019**, 9, 1901687.

[8] Y. Wang, C. Wang, X. Song, M. Huang, S. K. Megarajan, S. F. Shaukat, H. Jiang, *J. Mater. Chem. A* **2018**, 6, 9874.

[9] W. Xu, X. Hu, S. Zhuang, Y. Wang, X. Li, L. Zhou, S. Zhu, J. Zhu, *Adv. Energy Mater.* **2018**, 8, 1702884 .
[10] Q. Chen, Z. Pei, Y. Xu, Z. Li, Y. Yang, Y. Wei, Y. Ji, *Chem. Sci.* **2018**, 9, 623.
[11] Z. Guo, G. Wang, X. Ming, T. Mei, J. Wang, J. Li, J. Qian, X. Wang, *ACS Appl. Mater. Inter.* **2018**, 10, 24583.
[12] M. Ye, J. Jia, Z. Wu, C. Qian, R. Chen, P. G. O'Brien, W. Sun, Y. Dong, G. A. Ozin, *Adv. Energy Mater.* **2017**, 7, 1601811.
[13] H. Ren, M. Tang, B. Guan, K. Wang, J. Yang, F. Wang, M. Wang, J. Shan, Z. Chen, D.
Wei, *Adv. Mater.* **2017**, 29, 1702590.
[14] L. Huang, J. Pei, H. Jiang, X. Hu, *Desalination* **2018**, 442, 1.
[15] Y. Jin, J. Chang, Y. Shi, L. Shi, S. Hong, P. Wang, *J. Mater. Chem. A* **2018**, 6, 7942.
[16] Y. Kuang, C. Chen, S. He, E. M. Hitz, Y. Wang, W. Gan, R. Mi, L. Hu, *Adv. Mater.* **2019**, 31, e1900498.
[17] X. Guo, H. Gao, S. Wang, L. Yin, Y. Dai, *Desalination* **2020**, 488, 114535.
